# Printable elastic conductors with a high conductivity for electronic textile applications

**DOI:** 10.1038/ncomms8461

**Published:** 2015-06-25

**Authors:** Naoji Matsuhisa, Martin Kaltenbrunner, Tomoyuki Yokota, Hiroaki Jinno, Kazunori Kuribara, Tsuyoshi Sekitani, Takao Someya

**Affiliations:** 1 Electrical and Electronic Engineering and Information Systems, The University of Tokyo, 7-3-1 Hongo, Bunkyo-ku, Tokyo 113-8656, Japan; 2 Advanced Leading Graduate Course for Photon Science (ALPS), 7-3-1 Hongo, Bunkyo-ku, Tokyo 113-8656, Japan; 3 Exploratory Research for Advanced Technology (ERATO), Japan Science and Technology Agency (JST), 7-3-1 Hongo, Bunkyo-ku, Tokyo 113-8656, Japan; 4 The Institute of Scientific and Industrial Research (ISIR), Osaka University, 8-1 Mihogaoka, Ibaraki, Osaka 567-0047, Japan

## Abstract

The development of advanced flexible large-area electronics such as flexible displays and sensors will thrive on engineered functional ink formulations for printed electronics where the spontaneous arrangement of molecules aids the printing processes. Here we report a printable elastic conductor with a high initial conductivity of 738 S cm^−1^ and a record high conductivity of 182 S cm^−1^ when stretched to 215% strain. The elastic conductor ink is comprised of Ag flakes, a fluorine rubber and a fluorine surfactant. The fluorine surfactant constitutes a key component which directs the formation of surface-localized conductive networks in the printed elastic conductor, leading to a high conductivity and stretchability. We demonstrate the feasibility of our inks by fabricating a stretchable organic transistor active matrix on a rubbery stretchability-gradient substrate with unimpaired functionality when stretched to 110%, and a wearable electromyogram sensor printed onto a textile garment.

## Abstract (web summary)

Printable electronics is highly desirable for high throughput device manufacture. Here, Matsuhisa *et al*. report an electric ink, made of a self-assembled network of sliver flakes on the surface of a fluorine rubber matrix, which exhibits high conductivity and mechanical durability to achieve this goal.

## Graphical abstract

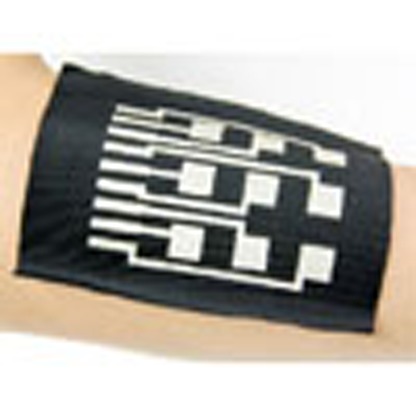

Printed electronics aim to minimize material waste and production costs, leading to reduced carbon dioxide emissions during manufacturing. This is especially significant for large-area electronics such as flat-panel displays^1^, photovoltaics^2^ and large-area sensors^3^,^4^. The formulation of next-generation electronic functional inks is essential to achieve these goals. Various kinds of inks ranging from conductors, insulators and semiconductors with inorganic and/or organic materials have been developed and utilized for printing transistors^5^, light-emitting diodes^6^ and solar cells^7^.

Due to recent intensive efforts, the resistivity of metallic inks has been reduced significantly, and Ag nanoparticle-based inks with resistivities of 2–3 μΩ cm, only 25–88% larger than bulk Ag, have been demonstrated at curing temperatures of over 150 °C (ref. 8). Although the calcination temperature can be lowered down to room temperature, the resistivity of these inks is still significantly higher than the bulk metal^9^. Lowering the process temperature can broaden the range of choices of substrate including low cost plastic films, rubber, textiles and even paper. In addition, solution processable semiconductors^10^,^11^,^12^,^13^ exhibit mobilities exceeding 20 cm^2^ V^−1^ s^−1^. This value is already exceeding that of amorphous Si, opening the door to many practical applications.

Printed electronics aim not only to replace individual electronic layers with cheaper alternatives, but also to enable the development of complex materials that deliver additional functionalities. For example, controlling the viscosity of a two component solution can be used to create a self-assembled structure based on the affinity of the solution components to each other. Phase separation has been used to create high mobility organic semiconductor channels wrapped in an insulating protective layer for driving circuits in a display^14^. Crystallization, phase separation and/or self-assembly of molecules can be controlled during the drying processes and utilized to obtain better electronic functionalities in a single processing step. In this way, a single print process can substantially simplify traditionally complex manufacturing processes.

An important design aspect for printed electronics is mechanical durability, especially for realizing flexible and/or soft and stretchable electronics. The formulation of stretchable and elastic conductors that can maintain their performance at ever increasing strain enables many new and important applications^15^,^16^,^17^,^18^. One of the most attractive targets is for the human body, which endures strains of 30% at skins^16^ and over 100% at joints^18^. Even commercially available sport wear is designed to be stretched by up to 400%. To apply printing processes to such applications, it is not sufficient to merely reduce process temperatures to be compatible with mechanically flexible substrates, as traditional conductors exhibit low strain-to-failure values of ∼1–2%. For example, delamination of printed electrodes from plastic films during flexing is a commonly observed problem that can be overcome by adding adhesive agents to the inks^19^. Mechanically robust electrodes, where high-aspect-ratio carbon nanotubes are uniformly dispersed in a rubber matrix as conductive fillers^15^,^20^, offer an alternative solution to realize elastic conductors.

In spite of intensive efforts, two major issues still remain for currently existing stretchable conductive materials: Due to the trade-off of their mechanical and electrical characteristics, increasing the conductive filler amount for higher conductivity increases the material stiffness which in turn diminishes the stretchability^15^,^20^. The implementation of nanometer-scale conductive networks can improve the mechanical properties, but requires multi-step fabrication processes making this approach distinct from a single-step printing process^21^,^22^. Developing a stretchable, conducting material that can be patterned in a single printing process with both high electrical and mechanical performance will thus fuel progress in printed electronics.

Here we report on the development of new electronic functional inks that simultaneously exhibit high conductivity and mechanical durability while being patternable in a single printing step. The conductivity of the elastic conductor is 738 S cm^−1^ (highest, average: 469±255 S cm^−1^) before stretching (strain of 0%) and remains as high as 182 S cm^−1^ at a strain of 215%; more than three times longer than its original length. The inks allow fine patterning at resolutions of 50 μm by stencil printing. The combination of conductivity and stretchability is enabled by self-assembled conductive networks of Ag flakes on the surface of a fluorine rubber matrix. The self-assembly is driven by the phase separation of a 4-methyl-2-pentanone and the water-based fluorine surfactant, increasing the stretchability through both the formation of a surface-localized Ag layer and plasticization of the polymer matrix. The feasibility of the elastic conductors is demonstrated by two applications: (1) a 110% stretchable organic transistor integrated circuit on a rubbery substrate with a stretchability gradient and (2) a wearable electromyogram (EMG) system on a conventional textile substrate.

## Results

### Highly stretchable elastic conductors

**Figure 1:**
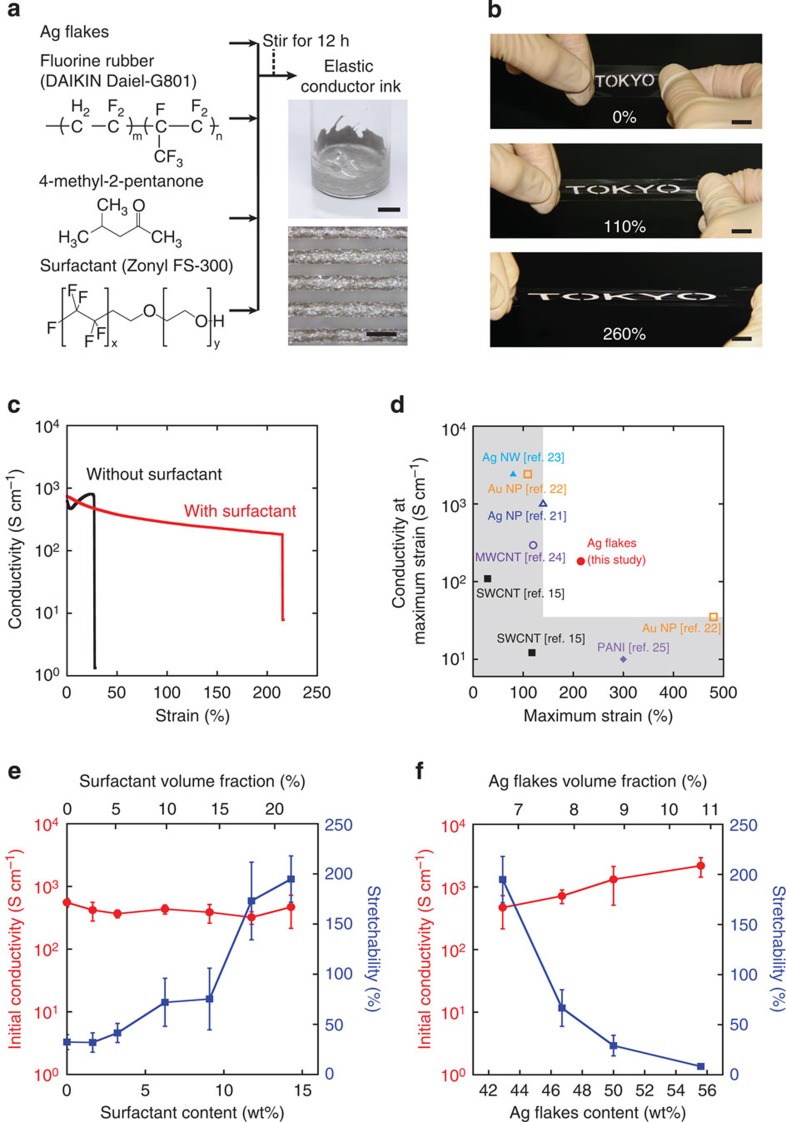
Highly stretchable elastic conductors. (**a**) Fabrication process of elastic conductor ink. Upper picture, elastic conductor ink. Scale bar, 10 mm. Lower picture, printed elastic conductor with high resolution. Scale bar, 100 μm. (**b**) Printed elastic conductor and demonstration of the stretchability. Scale bar, 10 mm. (**c**) Conductivity dependence on tensile strain of printed elastic conductor with and without surfactant. The maximum stretchability of elastic conductor with surfactant is limited by the strain limit of the substrate. (**d**) A comparison of this work to recent work in elastic conductors. Data points are extracted from the following papers: light blue filled triangle, Ag nanowires (Ag NW)—the study by Xu and Zhu^23^ (calculated from resistance change under the assumption that the total volume does not change); orange open square, Au nanoparticles (Au NP)—the study by Kim *et al*.^22^; blue open triangle, Ag nanoparticles (Ag NP)—the study by Park *et al*.^21^; purple open circle, multi walled carbon nanotubes (MWCNT)—the study by Chun *et al*.^24^; black filled square, single walled carbon nanotubes (SWCNT)—the study by Sekitani *et al*.^15^; light purple filled diamond, polyaniline (PANI)—the study by Stoyanov *et al*.^25^; red filled circle, this study (corresponds to **c**). (**e**) Initial conductivity and stretchability dependence on surfactant content. The weight ratio of Ag flakes, fluorine rubber and 4-methyl-2-pentanone was fixed at 3:1:2 (volume fraction, 1:1.94:8.74). Red circles, initial conductivity; blue squares, stretchability. (**f**) Initial conductivity and stretchability dependence on the Ag flakes content. The weight ratio of fluorine rubber, 4-methyl-2-pentanone and surfactant solution was fixed to 1:2:1 (volume fraction, 1:4.5:1.64). Error bars in **e**,**f** represent standard error.

The elastic conductor ink is prepared by adding Ag flakes (∼3.4 μm) as conductive fillers to an elastomeric fluorine copolymer (DAI-EL G801) with 4-methyl-2-pentanone as an organic solvent, together with a water-based fluorine surfactant (Zonyl FS-300, hereafter referred to as surfactant) (Fig. 1a). The size distribution of the Ag flakes, as obtained from Sigma Aldrich, is shown in Supplementary Fig. 1. The addition of surfactant improves the affinity of Ag flakes to the fluoropolymer matrix by modifying the Ag surface and by plasticizing the fluorine copolymer. This is discussed in more detail later in the manuscript. The conductive ink can be readily patterned with conventional printing techniques such as stencil printing or dispensers at high resolutions (50-μm line width, Fig. 1a) and into arbitrarily shaped, highly stretchable wirings like the 260% stretchable logo in Fig. 1b. The mixing ratio of the four ink components is a key parameter to control the electrical and mechanical properties of the resulting elastic conductors. Careful optimization of the mass ratio between Ag flakes:fluorine rubber:4-methyl-2-pentanone:surfactant to 3:1:2:1 results in the simultaneously highest conductivity and stretchability. The elastic conductor inks are patterned on 150-μm thick polydimethylsiloxane (PDMS) substrates by stencil printing to evaluate their electrical and mechanical characteristics (see also Methods and Supplementary Figs 2 and 3). PDMS is chosen as a supporting substrate due to its ease of processing and tunable Young's modulus. Comparing the conductivities of elastic conductors with and without surfactant while undergoing uniaxial tensile strain (Fig. 1c) shows that the initial conductivity (at zero strain) of the printed conductor containing surfactant is as large as the reference without surfactant and reaches 738 S cm^−1^. Most strikingly, introducing surfactant to the ink dramatically improves the stretchability (maximum strain before failure) of the elastic conductor, from a modest 27% for the reference to over 200% for the surfactant containing formulation. Stretchable forms of LED lightning and other high power electronics will greatly benefit from such high conductivity and stretchability, a combination of properties notoriously difficult to achieve. The conductivities at the maximum sustainable strain for state-of-the-art solution-processed stretchable conductors^15^,^21^,^22^,^23^,^24^,^25^ are compared in Fig. 1d. The conductivities as a function of strain are also compared in Supplementary Fig. 4. Our printed conductors reach a conductivity of 182 S cm^−1^ at a strain of 215%, currently the highest value reported for stretchable conductors that can be stretched >150%. Remarkably, device failure above strains of 200% always occurs due to rupturing of the PDMS substrate, not due to loss of conductivity or mechanical failure of the printed conductor itself. This suggests that further improvements are feasible by using more mechanically robust substrates which have an appropriate modulus and good adhesion with the printed elastomer. Porous stretchable substrates like polyuretahne foam or stretchable substrates that can be slightly dissolved with 4-methyl-2-pentanone are promising candidates.

The conductor's electrical and mechanical properties are sensitive to the weight ratio of the constituent materials. Thus, to understand the effect of each component on the conductor's electrical and mechanical properties, the concentration of each component is systematically changed while keeping the others fixed. We first evaluated the influence of the surfactant by increasing the concentration from 0 to 15 wt% (volume fraction, 0 to 21.4%) while the ratio of Ag flakes:fluorine rubber:4-methyl-2-pentanone is kept at 3:1:2 (volume fraction, 1:1.94:8.74) (Fig. 1e). The stretchability is dramatically improved from 32 to 194% with an increasing amount of surfactant, while the initial conductivity remains unchanged. We then increase the Ag flake content from 43 to 56 wt% (volume fraction, 6.7 to 10.7%) (Fig. 1f). As expected, the initial conductivity of the elastic conductors increases from 469 to 2,182 S cm^−1^. However, with a higher loading of Ag flakes, the maximum stretchability decreases from 194 to 8%. The surfactant used in this study is a formulation of 40 wt% solids in water and the effects of changing the surfactant–water concentration are presented in Supplementary Fig. 5a. Interestingly, at zero surfactant concentration (only pure water), the conductivity is as high as 2,180 S cm^−1^, while the stretchability is only 12%. Finally, we vary the concentration of 4-methyl-2-pentanone to evaluate how solvent evaporation affects the performance of the elastic conductors. The optimum conductivity is obtained at concentrations >28.6 wt% (volume fraction: 58.8%) 4-methyl-2-pentanone (Supplementary Fig. 5b).

The elastic conductor ink is viscous and is suitable for stencil printing, dispenser printing and screen printing. It can be printed at high resolution by a simple stencil printing and a mild annealing process at 80 °C to remove the solvent. Fine patterns with line width and pitch of 50 μm are obtainable on PDMS substrates (Fig. 1a) using a metal stencil mask. These lines exhibit a conductivity of 300 S cm^−1^. The conductivity of the thin lines may be lower from the different drying rate, accelerated by the high resolution of the printed line.

The performance under biaxial strain (0–93%) and twist angle (0–1,800°) are also evaluated (Supplementary Figs 6 and 7). The biaxial strain test shows that the elastic conductor keeps its conductivity even after 93% strain. In addition, under twisting, the elastic conductor's conductivity remains stable even after 1,800° of rotation. The endurance of the elastic conductor's electrical and mechanical properties is also assessed with cyclic fatigue tests up to 70% tensile strain. The cyclical endurance at 10% and 30% strain shows a resistance increase but the electrical conductivities do not diminish significantly even after 10^2^ cycles. However, at 50% and 70% strain a rapid loss of conductivity is observed (Supplementary Fig. 8). In this system, adhesion between the elastic conductor and the PDMS substrate^15^ seems to be one of the primary limiting factors for cyclic endurance. As shown in Supplementary Fig. 9, delamination of the elastic conductor is observed after the devices are strained at 100% for 10 cycles. This issue could be solved by adding cross-linking agents to the elastic conductor ink^20^, chemical modification with plasma or silane^26^ or top coating of elastic conductors with other elastomers^15^,^20^.

### Mechanisms of high conductivity and stretchability

Our highly conductive, highly stretchable conductors are obtained in a one-step, simple printing process. Triggered by the surfactant, phase separation and molecular self-assembly aid the formation of a Ag-rich, highly conducting layer near the surface of the printed elastic conductor. During this process the elastomer is plasticized, improving its mechanical properties. The combination of these two effects results in an elastic conductor with unprecedented stretchability and conductivity.

**Figure 2:**
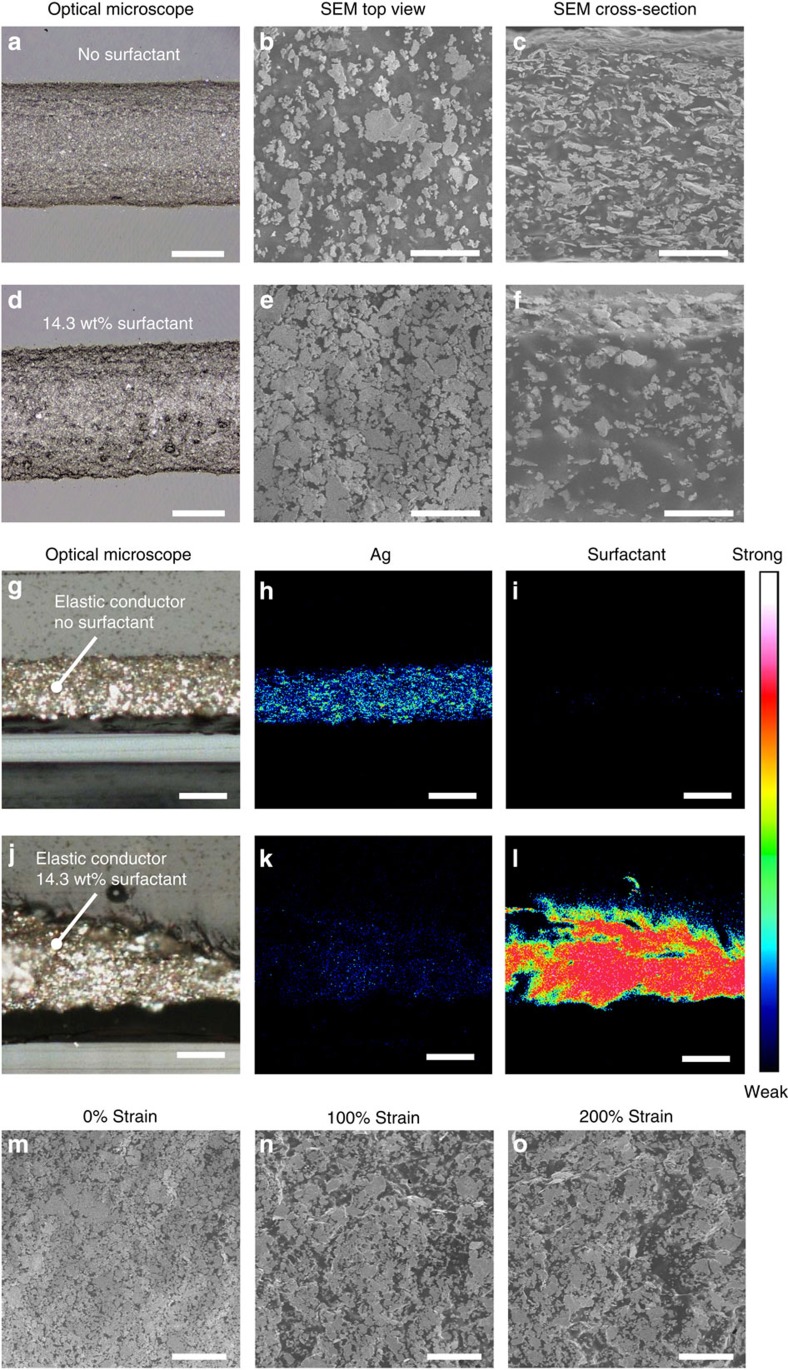
Elastic conductor self-assembly by phase separation. (**a**–**f**) Addition of surfactant/water solution results in a phase-separated morphology consisting of an elastic core topped by an Ag-dense surface layer. Optical microscopy and SEM images. In SEM images, bright areas correspond to Ag-rich phases, and the dark areas to non-conductive elastomeric regions. Upper row (**a**–**c**) without surfactant. Lower row (**d**–**f**) with surfactant. Left column (**a**,**d**) optical micrographs. Scale bars, 200 μm. Middle column (**b**,**e**) top-surface SEM images. Scale bars, 10 μm. Right column (**c**,**f**) cross-sectional SEM image. Scale bars, 10 μm. (**g**–**l**) Cross-sectional ToF-SIMS images. Upper row (**g**–**i**) without surfactant. Lower row (**j**–**l**) with surfactant. Left column (**g**,**j**) optical micrographs corresponding the ToF-SIMS images. Middle column (**h**,**k**) ToF-SIMS images of Ag. Right column (**i**,**l**) ToF-SIMS images of surfactant. Scale bars, 200 μm. Addition of surfactant reduces Ag signal as the surfactant binds to the Ag surface and is responsible for increasing the affinity between Ag flakes and fluorine rubber matrix. (**m**–**o**) Top-surface SEM images of stretched elastic conductors. (**m**–**o**) are elastic conductors stretched by 0%, 100% and 200%, respectively. Scale bars, 20 μm.

We characterize these multilayer structures by optical microscopy, scanning electron microscopy (SEM) and cross-sectional time-of-flight secondary ion mass spectroscopy (ToF-SIMS) to better understand the self-assembly process and to determine the distribution of Ag flakes and surfactant within and at the surface of the elastic conductor. Adding surfactant to the ink visually alters the surface of the printed structures, as observed by optical microscopy (Fig. 2a,d).

SEM images of both the top surfaces and cross-sections are shown in Fig. 2b,c,e,f, where the bright areas correspond to Ag-rich phases, and the dark areas to non-conductive elastomeric regions. Ag flakes are uniformly dispersed for the sample without surfactant, while Ag aggregates near the top surface when surfactant is added. After adding the surfactant, the surface density of Ag flakes increases. The surface modification of Ag flakes is supported by highly surface sensitive (1–2 nm penetration depth) cross-sectional ToF-SIMS analysis (Fig. 2g–l). The sample containing surfactant shows only a weak intensity for Ag (Fig. 2k) and a much stronger C_2_H_5_O intensity (Fig. 2l) associated with fluorine surfactant than the reference sample without surfactant (Fig. 2h,i). This suggests that the Ag flakes are coated with surfactant throughout the sample. Nonetheless, a high conductivity can still be maintained due to surface localization of Ag flakes, even while some Ag flakes appear to be insulated by the fluorine surfactant.

The SEM images of related experiments, showing the surface of elastic conductors without water and with different ratios of 4-methyl-2-pentanone are presented in Supplementary Figs 10 and 11, respectively. From these data, the phase separation^27^ of water and 4-methyl-2-pentanone seems crucial for obtaining a high density of Ag flakes on the surface of the conductor. In the absence of water, the Ag flakes are homogeneously dispersed on the surface of the elastic conductor (Supplementary Fig. 10). However, this condition showed no conductivity, a consequence of the poor networking on individual clusters of Ag flakes. Next, by varying the amount of 4-methyl-2-pentanone added, a drastic change in the surface composition can be observed (Supplementary Fig. 11). As the 4-methyl-2-pentanone concentration is increased, the density of Ag flakes on the surface also increases. The electrical conductivities follow the trend of the Ag flake surface density and is shown in Supplementary Fig. 5b. These observations demonstrate the importance of the interplay between water, surfactant and 4-methyl-2-pentanone in achieving high densities of Ag flakes on the surface of the elastic conductor.

In this work, highly stretchable and highly conductive materials are realized by choosing Ag flakes as conducting fillers despite the fact that high-aspect-ratio conducting materials such as carbon nanotubes^15^,^20^ and Ag nanowires^23^,^28^ are usually preferred due to their low percolation thresholds. To investigate why relatively low-aspect-ratio Ag flakes exhibit stable conductivities under strain, we characterize the elastic conductor under strains of 0, 100 and 200% by SEM (Fig. 2m–o). SEM images at lower magnification are available in Supplementary Fig. 12. The highly conductive surface layer forms cracks with increasing strain; however, a large number of percolation pathways are maintained even at strains of 200%. At low strains, the Ag-rich surface layers mainly contribute to the conductance. At high strain, the Ag flakes at the surface form cracks, but keeps enough highly conductive pathways. We hypothesize that the elastic conductor is readily compressed and maintains conductive paths in the core of the conductor, preserving high conductivity in this region of strain. We also expect strain-induced self-assembly^22^ of Ag flakes, and such phenomena would happen in the core where the density of fillers is low^29^. However, it is difficult to be observed because the surface is covered with high-density Ag flakes. Theoretical study might help understanding the physical process underlying this phenomenon.

### Stretchable organic transistor active matrix

**Figure 3:**
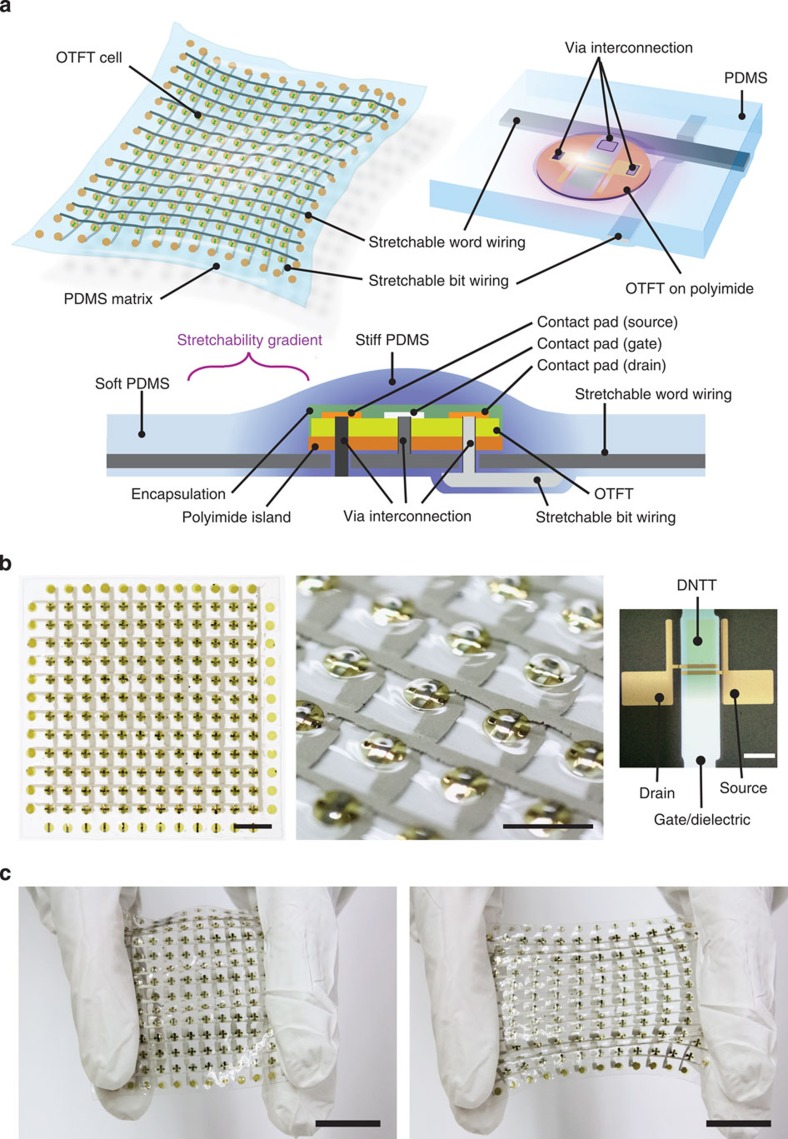
Soft and stretchable organic thin-film-transistor active matrix. (**a**) Illustrations of active matrix. Upper left, whole structure. Upper right, structure of an OTFT cell on a rigid island with elastic conductor wiring. Lower, schematic structure of an OTFT cell on the rigid island, the light green box corresponds to the entire OTFT device. (**b**) Pictures of OTFT-active matrix. Left, 12 × 12 active matrix. Scale bar, 10 mm. Centre, magnified OTFT cells. Scale bar, 5 mm; Right, optical micrograph of the embedded OTFT. Scale bar, 500 μm. (**c**) Active matrix is relaxed (left) and stretched to about 60% (right). Scale bars, 20 mm.

To demonstrate the potential of our printable elastic conductors for the design of large-area stretchable electronics, we choose two systems that fully utilize our inks. The first application is a stretchable organic transistor active matrix (Fig. 3). This integrated design is akin to stretchable devices reported by Rogers *et al*. but utilize organic transistors and printable elastic conductors, which are more scalable and mechanically robust than single-crystal Si and serpentine Au interconnects^16^. The 12 × 12 and 2 × 2 organic transistor active matrices are manufactured with soft, stretchability-gradient substrates (SGSs), in a hybrid rigid island-stretchable interconnect approach^30^,^31^,^32^,^33^. The diagram in Fig. 3a shows the design of the stretchable active matrix. In the SGS design, organic transistors are manufactured on non-stretchable, yet flexible, island-like regions. The organic transistor uses DNTT (dinaphtho[2,3-b:2′,3′-f]thieno[3,2-b]thiophene)^34^ as the active material, a high mobility (∼1–3 cm^2^ V^−1^ s^−1^) organic semiconductor. It has been shown to operate at low voltages and to be highly flexible^35^ and thermally stable^36^. The transistors are then embedded in PDMS sheets where wirings and interconnections are formed by printing elastic conductors. Completed devices, together with magnified views, are shown in Fig. 3b. As shown in Fig. 3c, the device is very soft (modulus: ∼0.5 MPa) and resilient to large deformations (∼60% strain). The detailed fabrication procedures are described in Methods, and Supplementary Fig. 13 (fabrication process of SGSs) and Supplementary Fig. 14 (print-wiring of elastic conductor).

**Figure 4:**
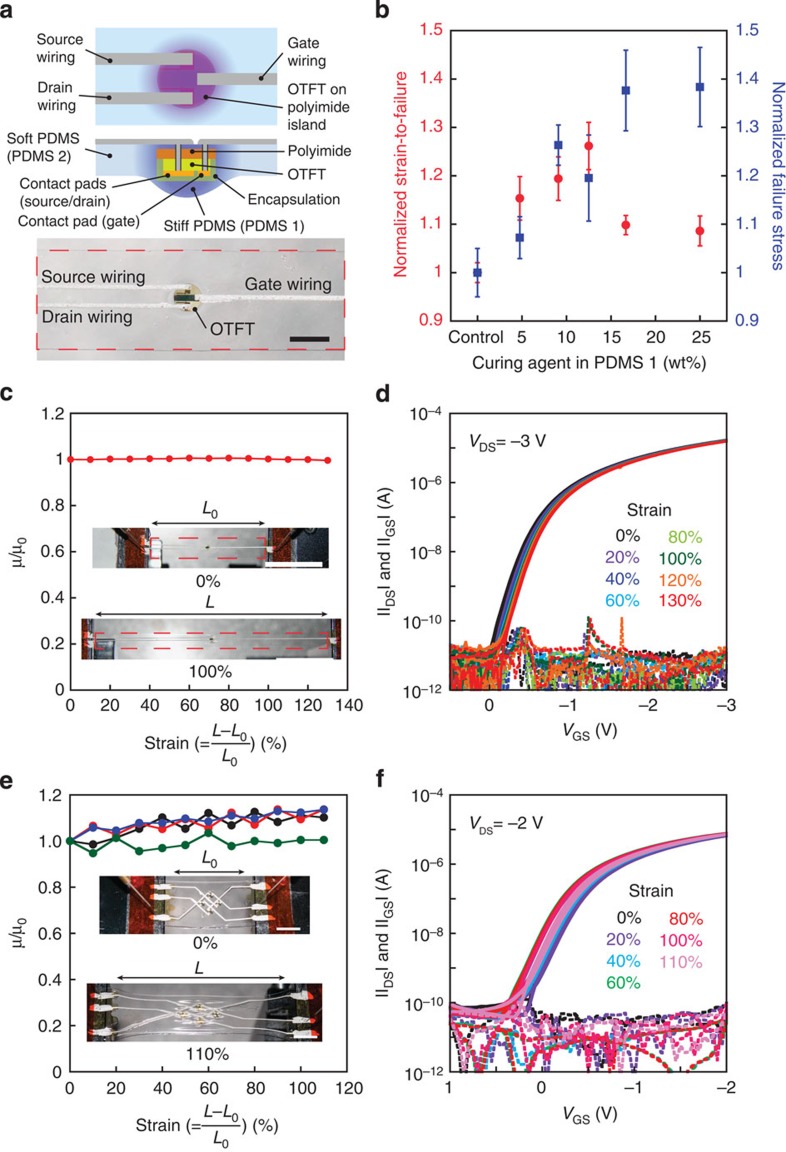
Stretchable organic transistors with stretchability-gradient substrate. (**a**) Device structure of a stretchable single transistor. Top, schematic from top surface. Centre, schematic from side, the green box corresponds to an entire OTFT device. Bottom, device picture. Scale bar, 5 mm. (**b**) Robustness evaluation with different concentrations of curing agent in PDMS 1. (PDMS 1 is patterned on stiff polyimide during the fabrication process of this substrate). Red circles, normalized strain-to-failure; blue squares, normalized failure stress. Error bars represent s.e. (**c**) Single stretchable transistor mobility dependence on tensile strain. Red circles represent normalized mobility. Scale bars, 3 cm. The inset shows the transistor relaxed (upper) and stretched (100%, lower). (**d**) Transfer characteristics of a relaxed and stretched single organic transistor corresponding to (**c**,**e**) mobility dependence on tensile strain of four transistors in a 2 × 2 stretchable active matrix. Scale bars, 1 cm. The inset shows the device in relaxed (upper) and stretched (110%, lower) configurations. (**f**) Transfer characteristics of one transistor in a stretchable active matrix corresponding to **e**.

With this design, a stretchable single transistor and active matrix are fabricated. Figure 4a shows the design of a stretchable single transistor. A transistor on a non-stretchable substrate is embedded in the stretchable PDMS and the three electrode pads are wired with the elastic conductor with a width and length of 500 μm and 3 cm, respectively. The Young's modulus and thickness of the PDMS layers are reduced gradually in expanding concentric circles from the rigid islands. To fabricate these structures, PDMS with a relatively high concentration (12.5 wt%) of curing agent (PDMS 1) is first patterned around stiff polyimide islands and then covered by PDMS with less (5 wt%) curing agent (PDMS 2). During the curing process, the curing agent diffuses^37^ and forms a gradient of stiffness.

Figure 4b shows the mechanical robustness with different curing agent concentrations (varied from 5 to 25 wt%) in PDMS 1. The device design for this evaluation is shown in Supplementary Fig. 15a. As a control sample, stretchable substrates without patterning of PDMS 1 (simply embedding polyimide islands in PDMS 2) are prepared. The SGSs with a curing agent concentration of 12.5% show the largest strain-to-failure ratio, 27% higher than the control devices. The failure stress increases as the concentration of curing agent in PDMS 1 increases. Next, elastic conductors with no surfactant (less stretchable) are patterned with the width of 500 μm and the thickness of 30 μm on SGSs and control substrates. Here improved performance of elastic conductor is expected because the strain would be uniformly dispersed in the stretchable region^30^. The resistance of the elastic conductor is evaluated with increasing the length of the device (Supplementary Fig. 15b). Elastic conductors on SGSs show a stretchability of 110%, while those on the control substrates sustain strains up to 50%.

The single transistor is manufactured on the SGS and connected with elastic conductors (Fig. 4c). The device is uniaxially stretched in steps of 10% strain and the transistor characteristics are measured at each step (Supplementary Fig. 16). The dependence of the mobility and the transfer curves on strain are shown in Fig. 4c,d, respectively. The mobility in the devices is 1.8 cm^2^ V^−1^ s^−1^, and is unchanged with increasing strain up to 130%. At 140% strain, delamination of the stiff polyimide islands is observed, leading to device failure.

We then fabricated 2 × 2 stretchable organic transistor active matrices and characterized their response to increasing strain. The whole system containing four transistors is stretched (up to 150%), and the change in mobility of the transistors is small up to strains of 110% (Fig. 4e). Figure 4f shows the typical transfer curves for one of the four transistors under different strains. All the transistors show stable characteristics at strains of ≤110%. At over 120% strain, two of the transistors exhibit irreversible degradation (Supplementary Fig. 17a), because one of the elastic conductors is torn (Supplementary Fig. 17b); however, the other two transistors exhibit reasonable thin-film-transistor (TFT) characteristics. At the strain of 150%, the device is torn at the edge of stiff polyimide island (Supplementary Fig. 17c).

### Textile EMG measurement system

**Figure 5:**
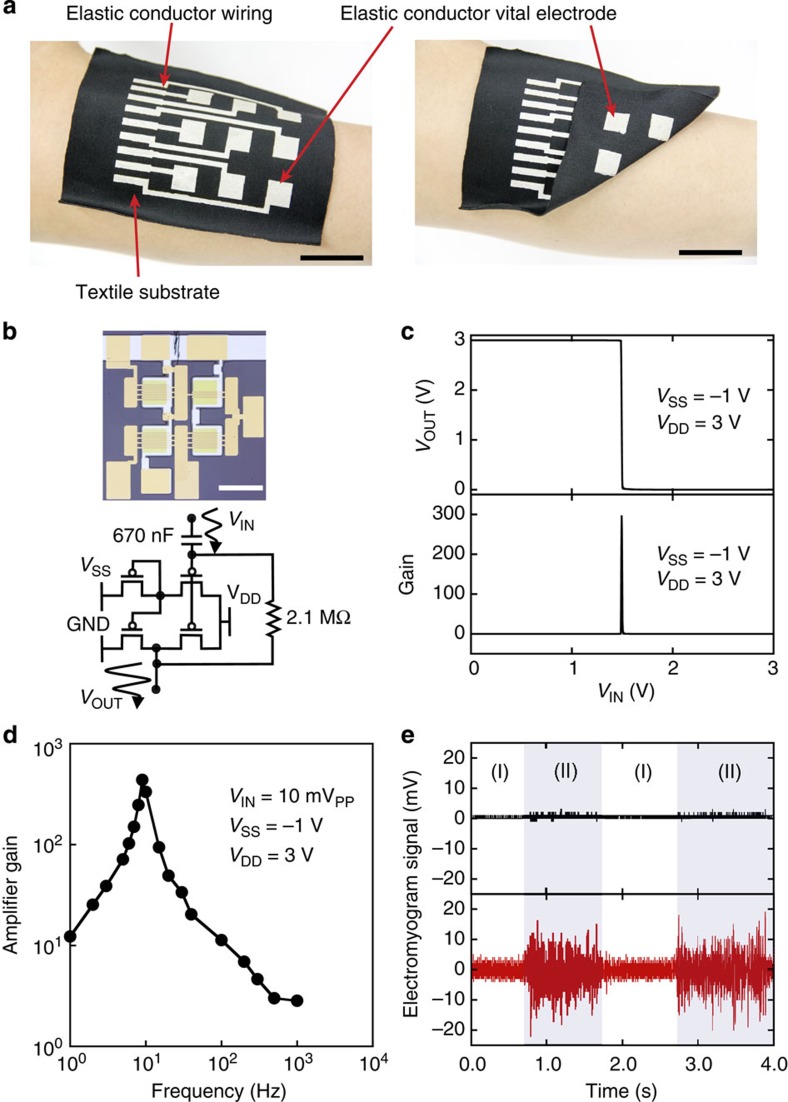
Measurement of arm EMG signals with an elastic conductor electronic textile. (**a**) Pictures of the EMG measurement system, using elastic conductor. Scale bars, 25 mm. (**b**) Organic amplifier circuit. Above, picture of organic amplifier circuit. Scale bar, 1 mm. Below, circuit diagram. (**c**) Performance of the organic pseudo-complementary metal–oxide–semiconductor inverter. Above, V_OUT_-V_IN_ curve. Below, inverter gain. (**d**) Frequency dependence of the amplifier gain. (**e**) EMG signals obtained through the elastic conductor attached to a forearm while opening (I) and closing a hand (II). Upper black line is the unamplified signal and lower red line is the signal amplified with the organic amplifier. Signals from muscle activity are observed when the hand is closed.

The second application explored is an e-textile or textile circuit board. We have realized an EMG measurement system, fully integrated on textiles, using our highly stretchable, highly conductive conductor. The detailed fabrication process is available in Methods. The elastic conductor can be printed on many unconventional substrates including textiles at room temperature; however, elevated temperatures may be used to reduce the vapourization time during the drying process. When printed on both sides of a stretchable cloth (Fig. 5a), the printed elastic conductors provide three main functionalities, namely, wiring on stretchy textiles, via interconnection and vital electrodes applicable to human skin. Water-proofing of devices, which is important when people sweat or wash devices, can be achieved by additional encapsulation layers formed by printing hydrophobic elastomers^38^.

Electrodes with an area of 20 × 20 mm^2^ are in direct contact with the human skin to obtain the EMG signal, which is typically in the voltage range of 1 mV. The signal is transmitted through via-holes and wirings with elastic conductors patterned on the cloth and then connected to a pseudo-complementary metal–oxide–semiconductor, organic transistor-based amplifier^39^. Figure 5b shows the circuit diagram and photograph. The inverter gain and amplifier spectrum can be seen in Fig. 5c,d, respectively. The maximum gain is as high as 290 and the cutoff frequency exceeds 1 kHz, which is sufficiently high for measuring EMG signals. The elastic conductor exhibits constant impedance up to 100 kHz (Supplementary Fig. 18). This is much higher than the bandwidth of the organic amplifier. An acrylamide adhesive gel electrode attached to skin serves as a ground electrode. The EMG signals are measured before and after amplification (Fig. 5e). Both of the data sets are filtered with a low pass filter with a cutoff frequency of 1 kHz. Then, the EMG signal is observed to synchronize with hand motions, and is amplified with the organic amplifier by a factor of 18. The study protocol was thoroughly reviewed and approved by the ethical committee of the University of Tokyo (approval number KE14-25).

## Discussion

The reason that enhanced conductivity and stretchability are simultaneously realized by the introduction of the fluorine surfactant can be summarized by three important factors. First, the surface aggregation of Ag flakes is induced by the self-assembly due to interaction between water, 4-methyl-2-pentanone and the fluorine surfactant. This surface aggregation allows for high conductivity even at low concentrations of Ag (volume fraction: 23.7%, without solvent), maintaining material softness. Second, the surface modification of Ag flakes by fluorine surfactant^22^,^40^ increases the affinity between the fluorine copolymer and conductive Ag flakes. Third, the plasticization of fluorine rubber increases the stretchability of the polymer matrix.

To simultaneously achieve large stretchability and large conductivity, we emphasize that the selection of fluorine rubber and surfactant is critical. Indeed, the combination of G801 and Zonyl FS-300 exhibits excellent compatibility. Besides G801, two other fluorine rubbers, namely, fluorine terpolymer (DAI-EL G912) and fluorine rubber specialized for low temperature (DAI-EL LT302) are tested to fabricate elastic conductors using the above optimized materials ratio for G801. However, only conductors employing G801 show excellent stretchability (Supplementary Fig. 19a). We then added surfactant to these fluorine rubbers to verify their compatibility with the surfactant. We found that only elastic conductors with G801 form uniform films (Supplementary Fig. 19b).

Finally, we would like to comment on several different approaches to introduce gradient stretchability to the rubber substrate. Compared with elastomers with a photo-controllable modulus^30^,^31^ and multi-layered structures of polymers with different moduli^32^, our method is the simplest process for the integration of multiple devices. By fabricating devices on a mechanically and chemically stable polyimide film and subsequently embedding in PDMS, device fabrication reproducibility on a stretchable substrate is significantly improved. Gradual changing of the substrate thickness has also been used for spatial modulation of stretchability^33^. However, large changes in substrate thickness introduce additional challenges for consistent device fabrication. Our approach also utilizes spatial change of modulus; however, sufficient durability could be realized without large thickness changes that would impede the device fabrication processes.

## Methods

### Materials and preparation of elastic conductor

Ag flakes were purchased from Sigma Aldrich (product number: 327077 Aldrich, Silver flakes, 10 μm, ⩾99.9% trace metals basis). The size distribution was measured by a laser diffraction particle size analyzer (SALD-2300, SHIMADZU Corp., Japan). Fluorine rubbers (DAI-EL G801, DAI-EL G912 and DAI-EL LT302) were received from DAKIN INDUSTIRIES, Ltd, Japan. The Ag flakes and fluorine surfactant/water solution (Zonyl FS-300) were purchased from Sigma Aldrich, USA. They were mixed with 4-methyl-2-pentanone as solvent by a magnetic stirrer for ⩾12 h in the abovementioned ratio. After printing, the films were heated at 80 °C for 30 min to remove excess solvent.

### Measurement and evaluation of elastic conductor

Supplementary Figure 2a shows the design of elastic conductors printed on silicone rubber using 125-μm-thick polyimide shadow masks. Stiff polyimide platforms were embedded in the silicone rubber with a stretchability gradient as explained later with Supplementary Fig. 13. The thickness and width of the printed elastic conductor were measured by laser microscope (VK-9710, KEYENCE Corp., Japan). The average values were 30-μm thick and 500-μm wide. The length was determined by the prepared substrate and was 3 cm. Next, the printed elastic conductors were stretched with a high-precision mechanical system (AG-X, SHIMADZU Corp.) with a stretching speed of 10% min^−1^ and the conductivity was measured using the four-probes-method using a digital multi-source meter (2400, Keithley Instruments Inc., USA) as shown in Supplementary Fig. 2b. Five samples were evaluated for each condition of the elastic conductor ink. The conductivity under strain was calculated from the measured geometry and resistance under the assumption that the total volume remained constant. This is reasonable considering that the Poisson ratio at 150% strain is 0.51±0.05, which was measured with a laser microscope by observing the same locations as shown in Supplementary Fig. 3. In addition, the conductivity was assumed to be uniform along the printed elastic conductor to compare our results with previous reports. Biaxial strain tests are performed by inflating PDMS balloons coated with the elastic conductor (Supplementary Fig. 6). The twisting behaviour was evaluated by using a clamp to maintain a constant lateral strain (0%) while twisting the device from one end (Supplementary Fig. 7). For cyclic endurance measurements, the surface of the PDMS substrate was exposed to UV-ozone (UV-1, SAMCO Inc., Japan) for 1 min before printing to improve the adhesion. The stretching and releasing speed was kept to 5% s^−1^. SEM images were taken by S4800, Hitachi High-Technologies with an accelerating voltage of 5 kV.

### Fabrication of organic thin-film-transistor

Aluminium (100-nm thick) was evaporated on 12.5-μm-thick polyimide film as a gate electrode. Metal shadow masks were used for patterning in all the vacuum evaporations. For the fabrication of gate dielectrics, a 19-nm-thick aluminium oxide was formed via anodic oxidation^41^. Subsequently, the surface was activated with oxygen plasma and then immersed in 5 mM octadecylphosphonicacid 2-propanol solution for 16 h to form a self-assembled monolayer^42^. Then, DNTT^34^ was evaporated as the semiconductor. Au was deposited with a channel length of 50 μm and channel width of 450 μm for the stretchable single transistor or 2,750 μm for the stretchable active matrix to form the source and drain electrodes. Finally, parylene (diX-SR, Daisan Kasei Co Ltd., Japan) was formed using chemical vapour deposition as an encapsulation layer with a thickness of 1.5 μm.

### Embedding organic thin film transistors in PDMS with stretchability gradient

Supplementary Figure 13 shows the process for embedding organic thin film transistors (OTFTs) with a stretchability gradient. First, the back side of the transistors was modified with oxygen plasma, followed by spin coating of a highly diluted silicone rubber liquid (Sylgard 184, Dow Corning Toray Corp., Japan; base:curing agent:hexane=10:1:20) at 6,000 r.p.m. for 1 min. Then, the device was annealed at 80 °C for 5 min to dry hexane and partially crosslink the silicone. This ∼1-μm-thick silicone layer worked as the adhesion layer. Next, devices were laminated on fluorine polymer-coated glass using the thin silicone layer, after creating through-holes to the devices for improving the adhesion. After heating at 80 °C for 5 min again, circular frames were cut around organic transistors with a diameter of 2.6 mm using a green laser (T-Centric SHG Laser Marker MD-T 1000, KEYENCE). These frames served as rigid islands. Next, silicone rubber liquid (Sylgard 184 base:curing agent=7:1) was spin coated at 500 r.p.m. for 1 min, followed by the delamination of films, leaving only circular-shape-cut transistors, which worked as rigid islands in the whole device. Then, we were able to pattern transistors and silicone rubber with higher curing agent content. After heating at 80 °C for 5 min, the silicone rubber liquid (Sylgard 184 base:curing agent=20:1) was spin coated on the entire device at 500 r.p.m., 1 min. Finally, the device was annealed at 80 °C for 2 h.

### Printing elastic conductors as wiring of stretchable transistors

Supplementary Figure 14 illustrates the printing process of the elastic conductor wires. First, an organic-transistor-embedded silicone rubber sheet was peeled from the fluorine polymer-coated glass and turned upside down onto the PEN film. Then, a silicone rubber liquid (Sylgard 184 base:curing agent=20:1) was spin coated at 4,000 r.p.m. for 1 min, followed by heating at 80 °C for 2 h. This layer prevented the short circuit of printed elastic conductors through the conductive laser-cut edge of the polyimide film. Then, laser-via-holes were made for the electrode pads of gate, source and drain of transistors. Next, elastic conductors were patterned as stretchable word lines and connected to gate electrodes through the holes. The thickness and width were ∼30 μm and 500 μm, respectively. Then, 100-μm-thick and 1-mm-wide insulating silicone rubber liquid (Sylgard 184 base:curing agent=20:1) was patterned with the stencil printing method, followed by heating at 80 °C for 2 h. Next, bit lines and ground lines were patterned with elastic conductor and connected to source and drain electrodes. Then, we obtained the stretchable organic transistor active matrix. The organic transistors were exposed to temperatures up to 80 °C during fabrication, well below their thermal stability of up to 110 °C (ref. 36).

### EMG device fabrication

First, laser-via-holes were made in a commercially available, stretchable sport textile (CW-X, Wacoal Corp., Japan). Then, the elastic conductor ink was printed with polyimide stencil masks on both sides of the textile, and the device was heated at 80 °C for 30 min after each printing step. The elastic conductors on one side are wirings and those on the other side are the vital electrodes for EMG recording. They are electrically connected through via-holes.

## 

## Additional information

**How to cite this article:** Matsuhisa, N. *et al*. Printable elastic conductors with a high conductivity for electronic textile applications. *Nat. Commun.* 6:7461 doi: 10.1038/ncomms8461 (2015).

## Supplementary Material

Supplementary Information Supplementary Figures 1-19
