# Supplementary material for: Printable elastic conductors with a high conductivity for electronic textile applications
Source: Nat Commun. 2015 Jun 25;6:7461. doi: 10.1038/ncomms8461 (PMC4491189; doi:10.1038/ncomms8461)
Supplement: Supplementary Information — Supplementary Figures 1-19 [file ncomms8461-s1.pdf]

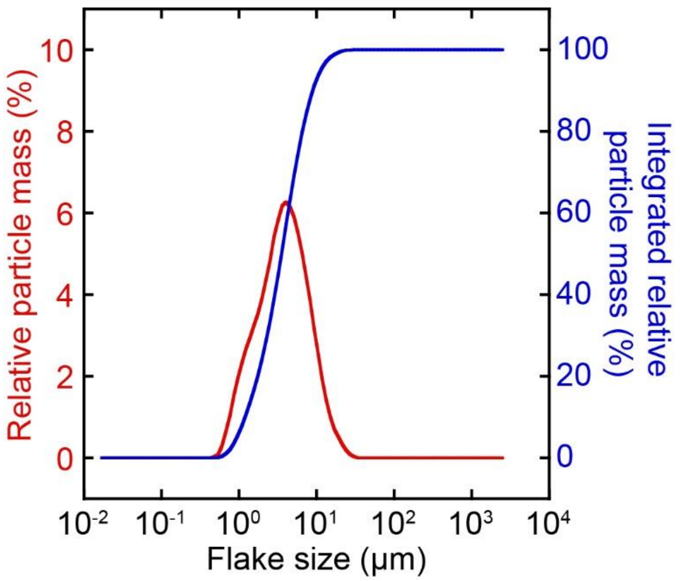

Supplementary Figure 1

Ag flake size distribution. The average size is 3.4 μm with standard deviation of 0.33 μm.

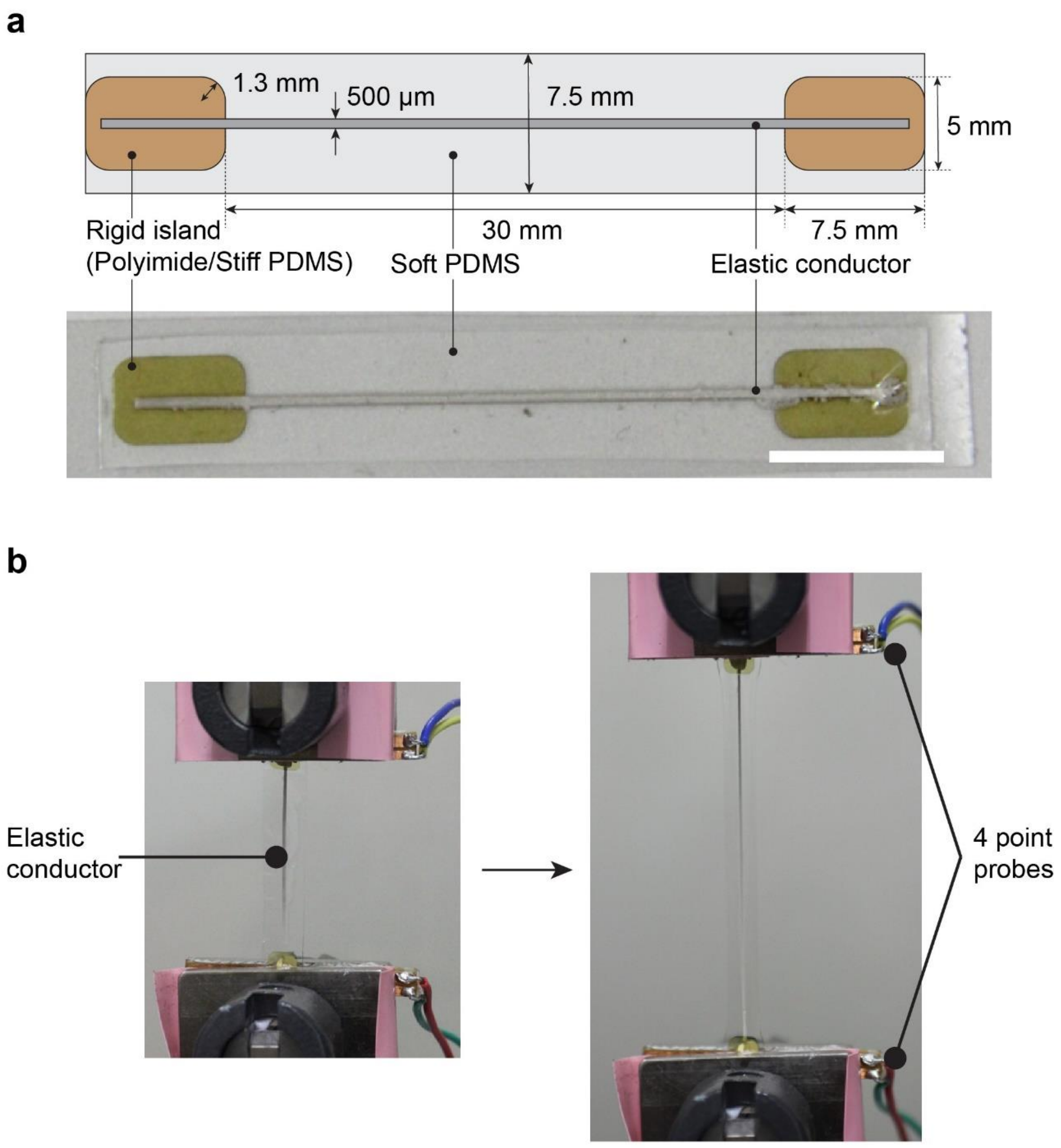

Supplementary Figure 2

Methods to evaluate elastic conductors.

a, Structure for the evaluation. The elastic conductor is printed on a stretchability gradient substrate. Scale bar, 1 cm. **b,** Measurement set up. The elastic conductor is stretched and electrically measured using the four-terminal method.

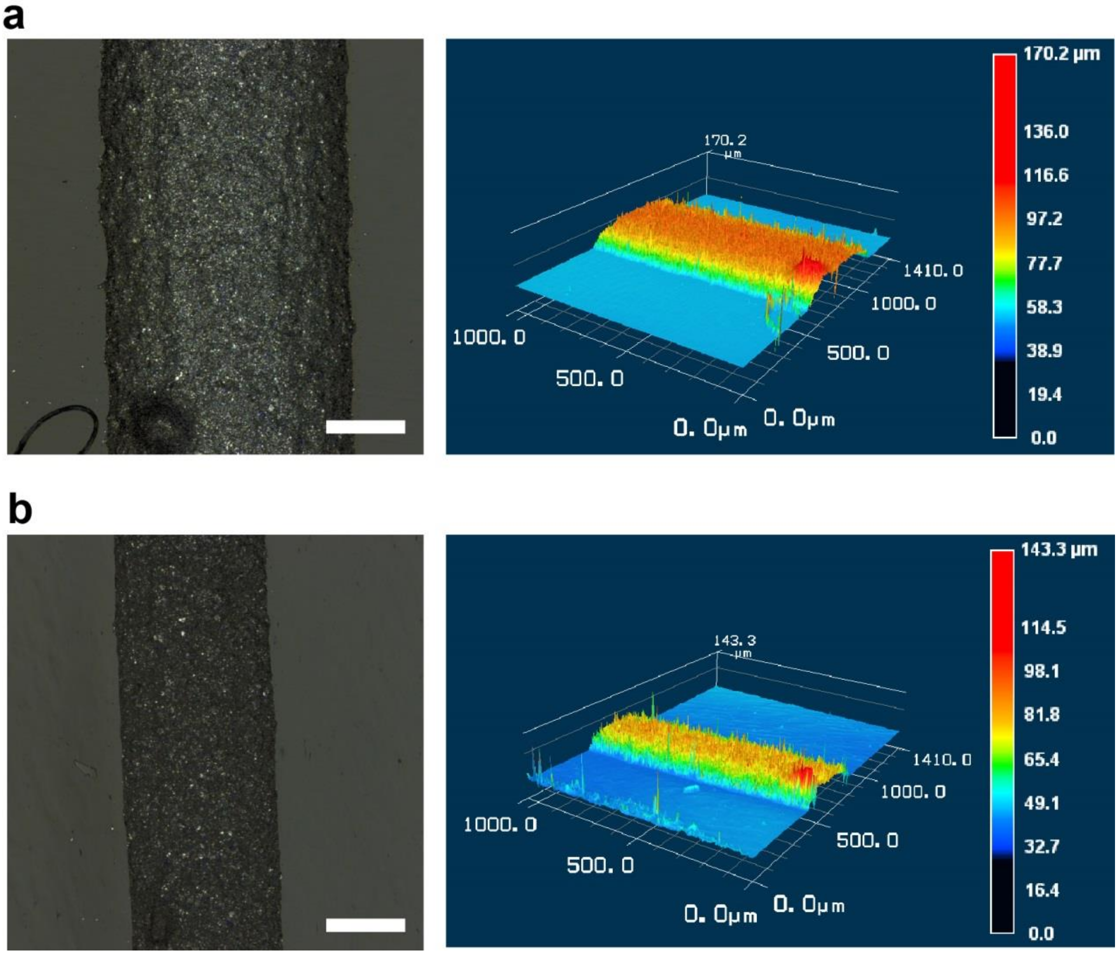

Supplementary Figure 3

Poisson ratio measurement of elastic conductor.

Laser microscope images of the elastic conductor **a,** without stretching and **b,** with stretching. Left, Optical images. Scale bars, 200 µm. Right, 3D images.

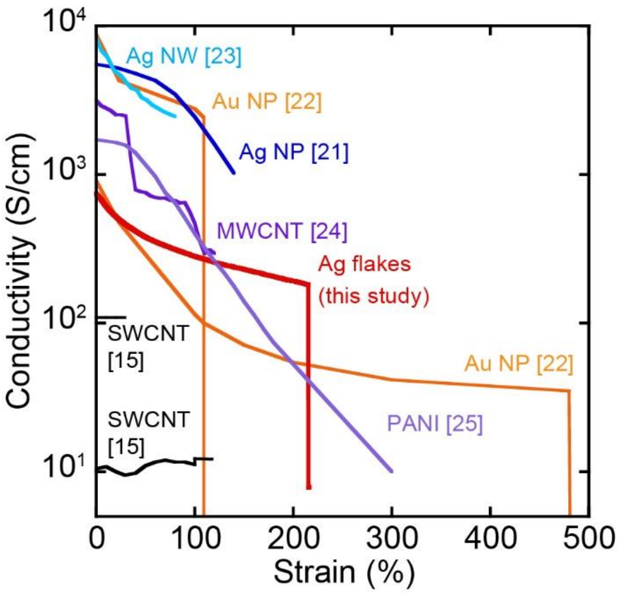

Supplementary Figure 4

A comparison of this work to literature. (Data points before rupture are also plotted.) Data points are extracted from the following papers: Light blue, Ag nanowires (Ag NW) [23] (calculated from resistance change under the assumption that the total volume does not change); Orange, Au nanoparticles (Au NP) [22]; Blue, Ag nanoparticles (Ag NP) [21]; Purple, multi walled carbon nanotubes (MWCNT) [24]; Black, single walled carbon nanotubes (SWCNT) [15]; Light purple, polyaniline (PANI) [25]; Red, this study (corresponds to Fig. 1c).

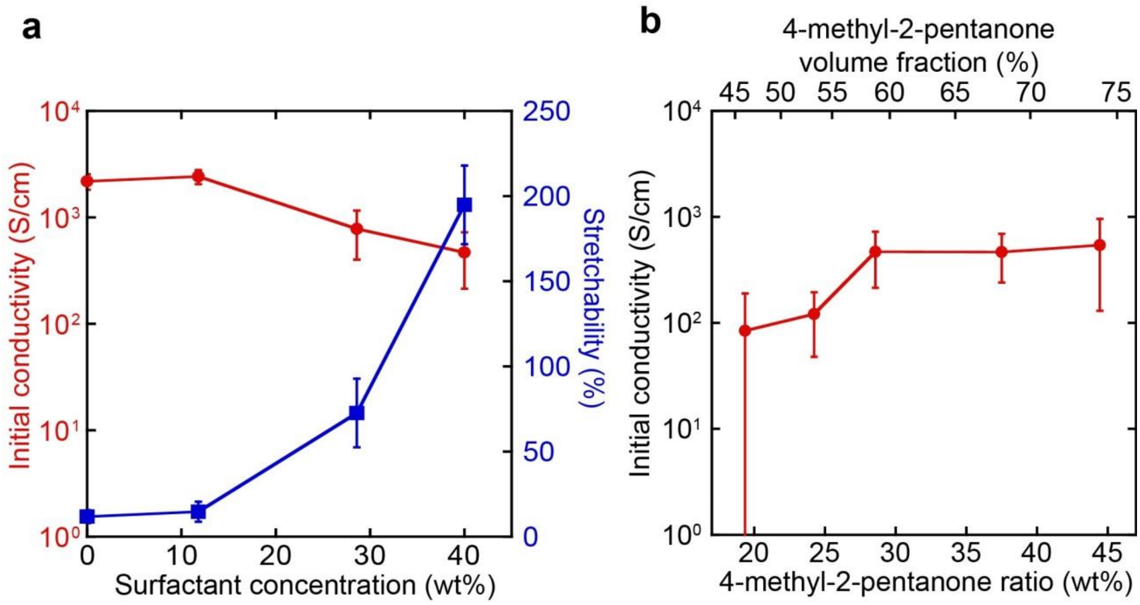

Supplementary Figure 5

Optimization of elastic conductors.

a, Initial conductivity and stretchability dependence on the surfactant concentration. The weight ratio of Ag flakes, fluorine rubber, 4-methyl-2-pentanone and water is fixed to 3:1:2:0.6 (volume fraction, 1:1.94:8.74:2.1). Red circle, initial conductivity. Blue square, stretchability. **b,** 4-methyl-2-pentanone content dependence on the initial conductivity of elastic conductor. The weight ratio of Ag flakes, fluorine rubber, and surfactant is fixed to 3:1:1 (volume fraction, 1:1.94:3.18). Error bars represent standard errors.

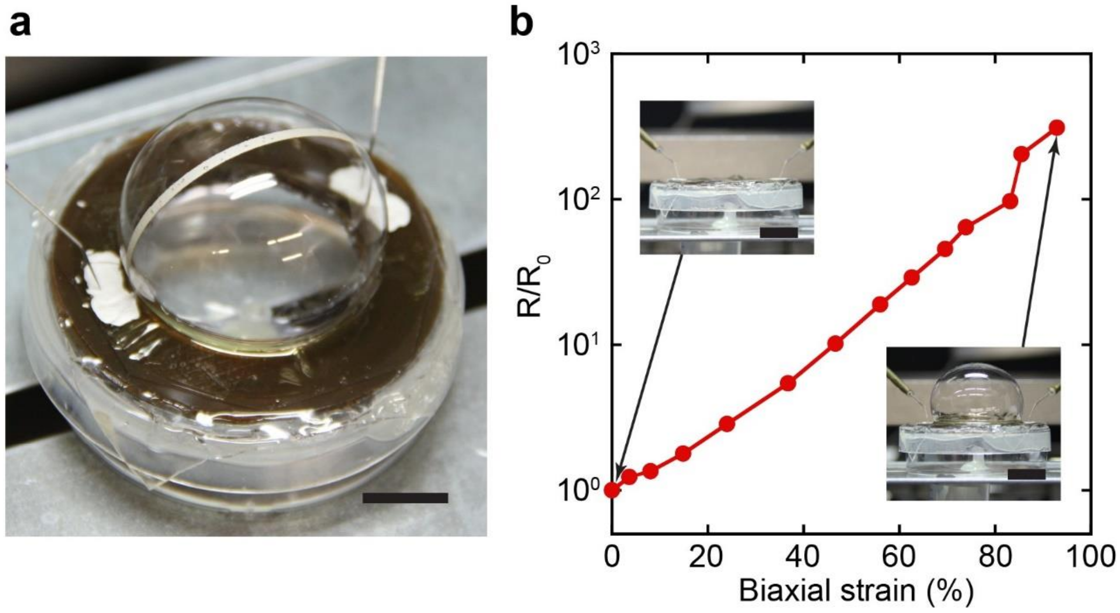

Supplementary Figure 6

Biaxial stress test of elastic conductor.

a, Biaxially stretched elastic conductor by an inflated PDMS substrate. Scale bar, 1 cm. **b,** Relative resistance change of the elastic conductor with biaxial strain. Scale bars, 1.5 cm.

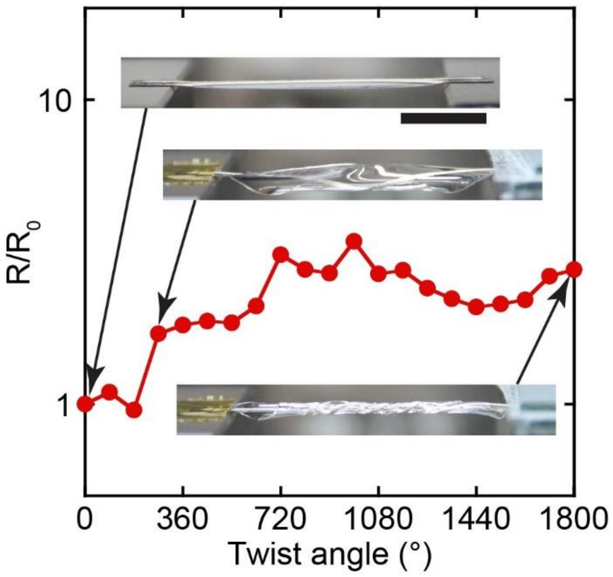

Supplementary Figure 7

Relative resistance change of elastic conductor during twisting. Scale bar, 1 cm.

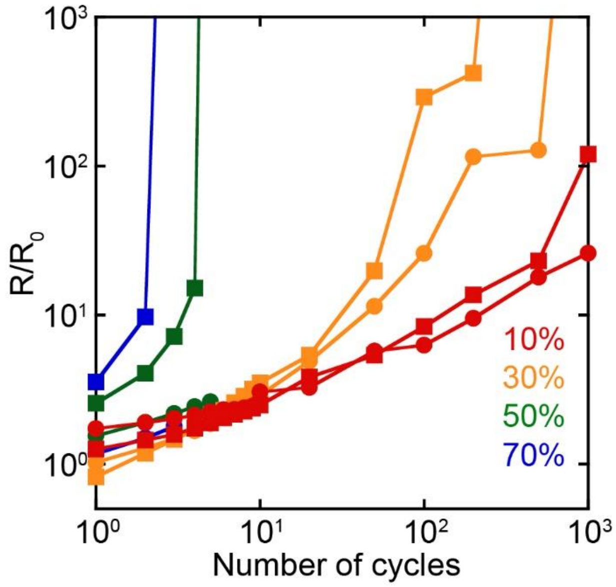

Supplementary Figure 8

Cyclic endurance of elastic conductor. 10%, 30%, 50%, and 70%-cyclic strain are applied to the elastic conductor. The resistance change is measured during stretch (square) and after stretch (circle).

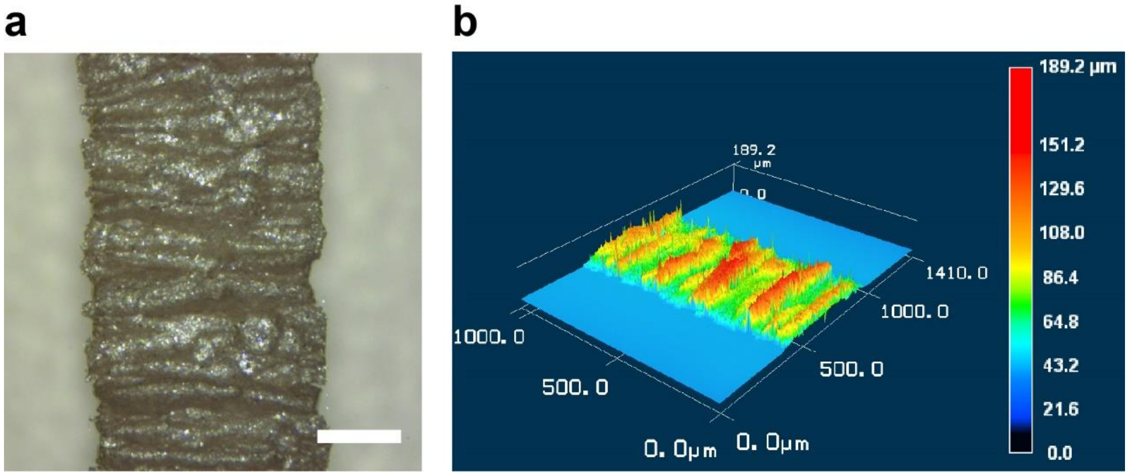

Supplementary Figure 9

The elastic conductor delaminated from PDMS substrate. a, Color laser microscope image. Scale bar, 200 µm. **b,** 3D profile. The pictures are taken after 10 cycles of 100% strain.

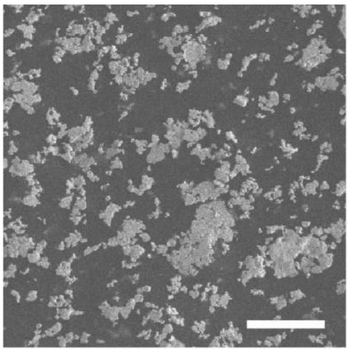

Supplementary Figure 10

Surface SEM images of elastic conductor without water. Water is eliminated from the optimized recipe (Ag flakes: fluorine rubber: 4-methyl-2-pentanone: surfactant = 3:1:2:1) when the ink is prepared. Scale bar, 10 µm.

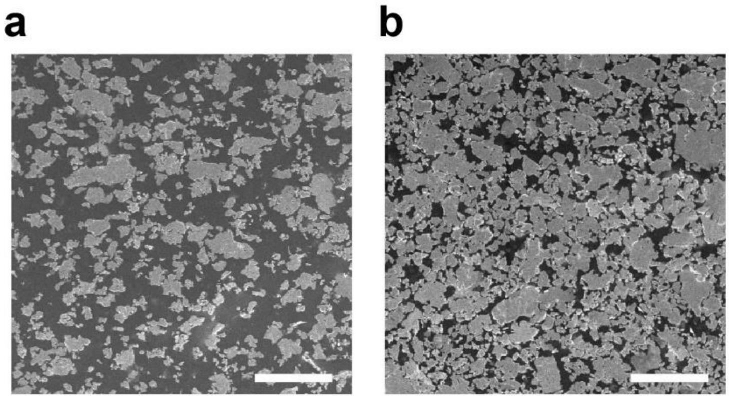

Supplementary Figure 11

Surface SEM images of elastic conductor with different concentrations of 4-methyl-2-pentanone. The weight ratio of Ag flakes, fluorine rubber, and surfactant is fixed to 3:1:1. **a,** 19.4 wt%. **b,** 37.5 wt%. Scale bars, 10 µm.

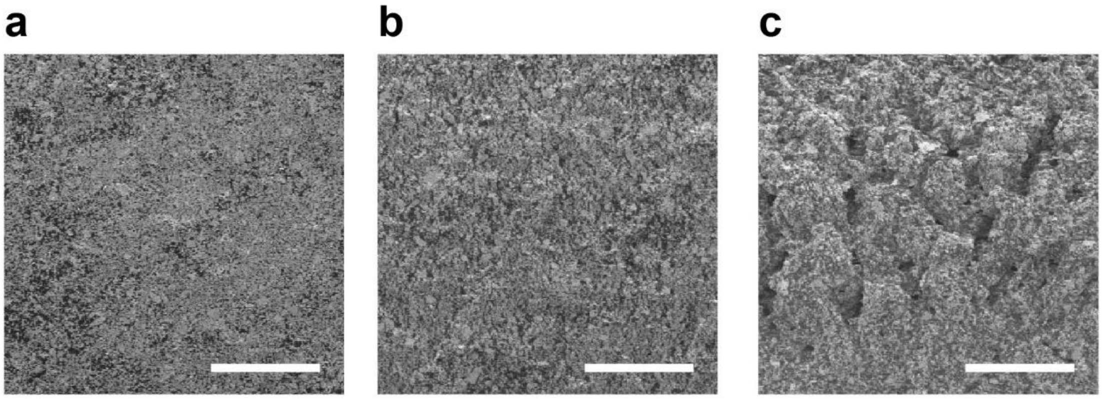

Supplementary Figure 12

Surface SEM images of elastic conductor with strain in lower magnification. a, 0% strain. **b,** 100% strain. **c,** 200% strain. Strains are applied in the lateral direction. Scale bars, 100 μm.

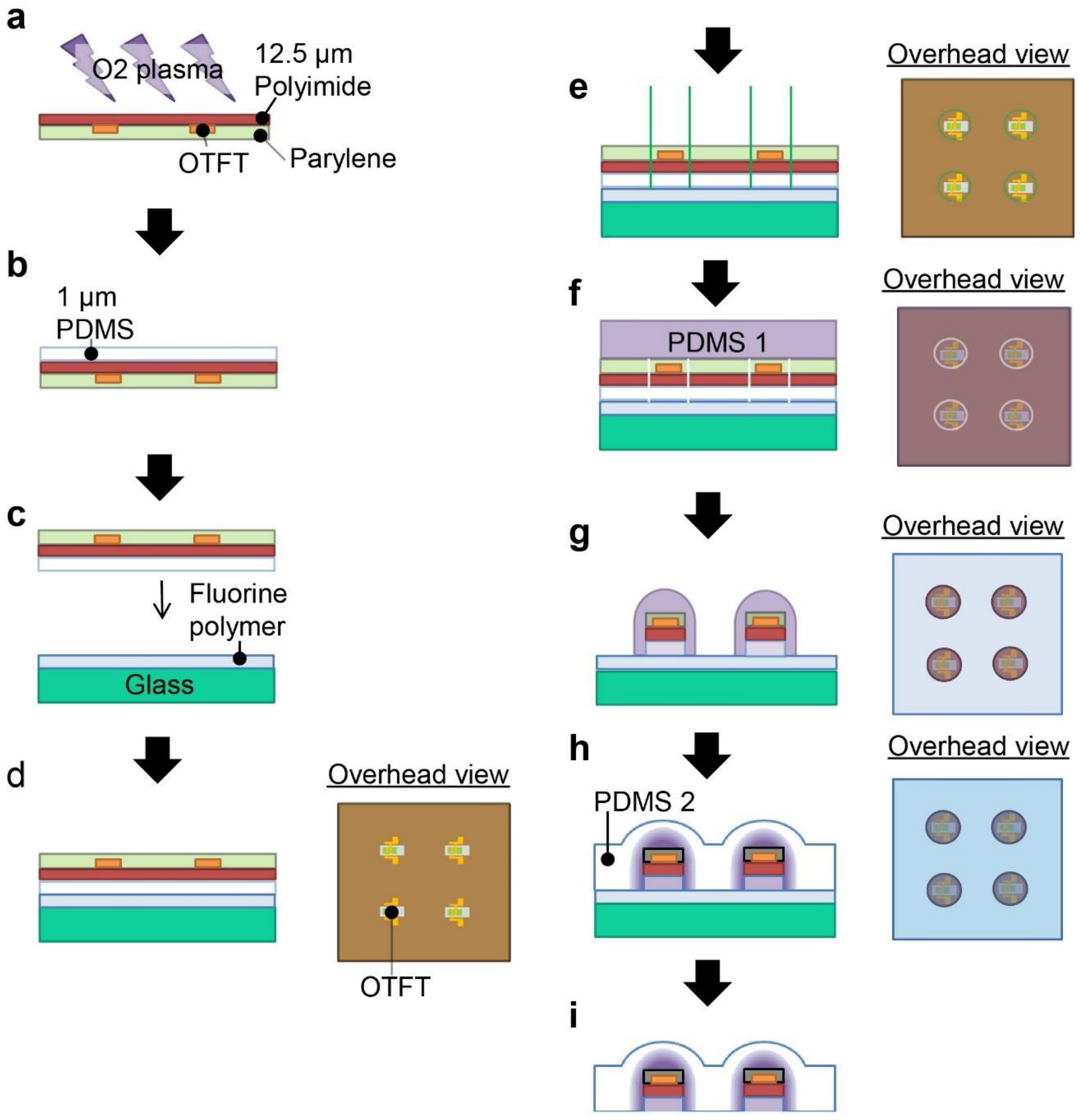

Supplementary Figure 13

Fabrication process of the stretchability gradient substrate (SGS). a, The back side of the transistors is modified with oxygen plasma. **b,** A highly diluted silicone rubber liquid is spin-coated. **c,d,** Devices are laminated on fluorine polymer coated glass. **e,** Circular frames are cut around organic transistors using a green laser. **f,** PDMS 1 is spin-coated. **g,** Extra part of films are delaminated and PDMS 1 is simultaneously patterned. **h,** PDMS 2 is spin-coated. **i,** Devices are delaminated from glass.

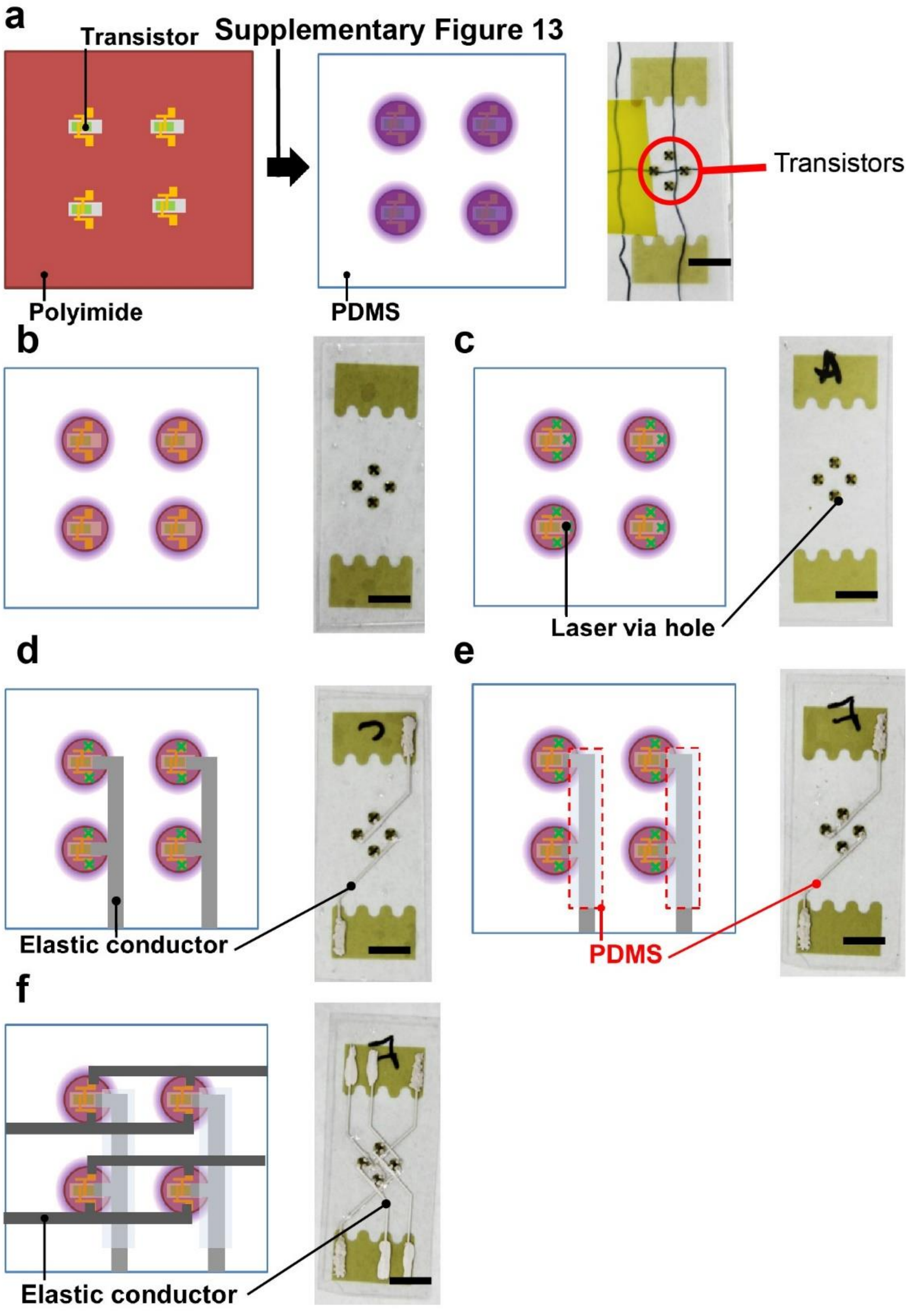

Supplementary Figure 14

Wiring organic transistors by printing elastic conductor and silicone rubber. Scale bars, 1 cm. **a,** OTFTs are embedded in PDMS. **b,** Devices are turned upside down and PDMS is spin-coated. **c,** Lase-via-holes are made to contact pads. **d,** Stretchable gate wirings are printed. **e,** PDMS insulator is printed. **f,** Stretchable source/drain wirings are printed.

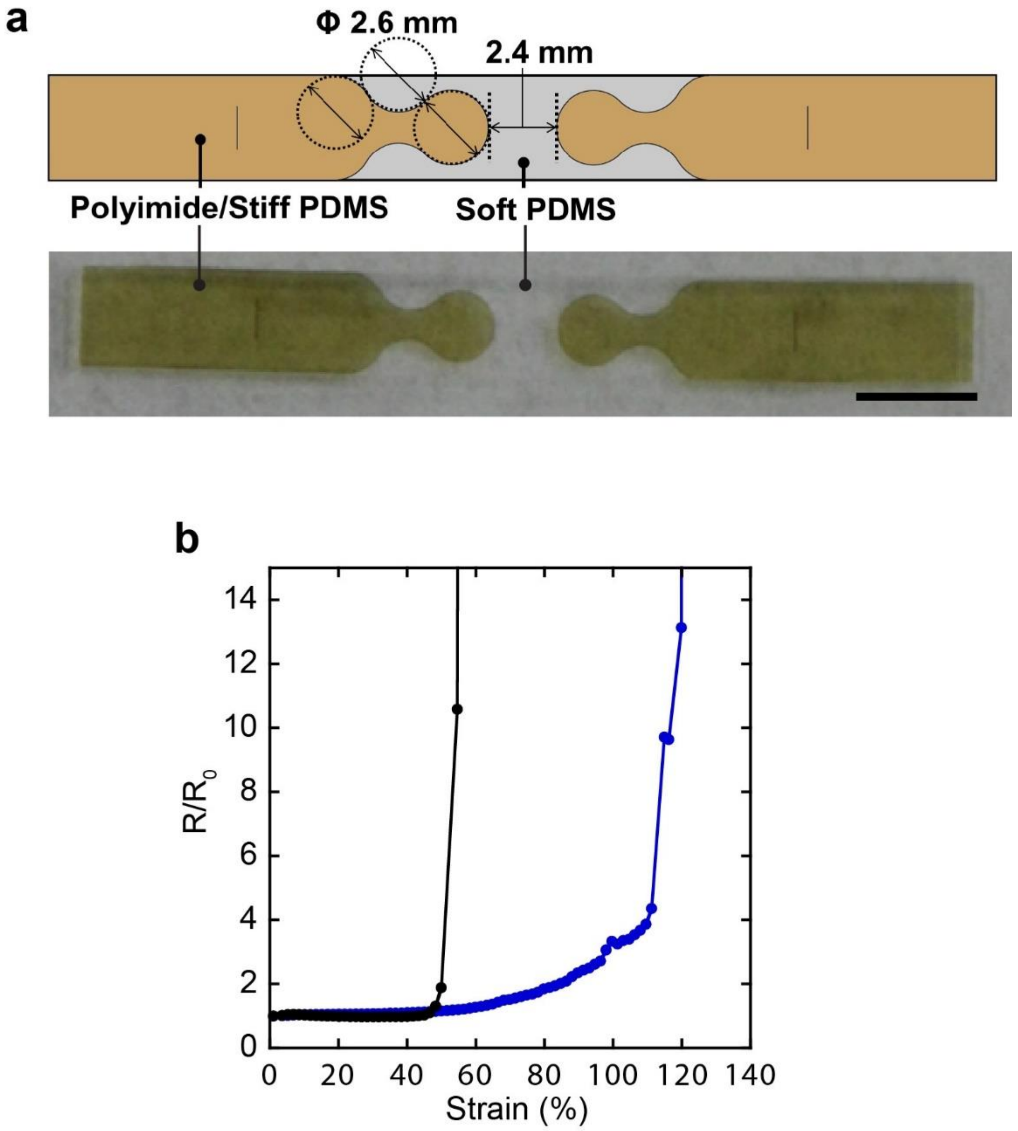

Supplementary Figure 15

Evaluation of SGSs. a, Structure for evaluation of stretchability-gradient-substrates. Scale bar, 5 mm. **b,** Resistance change of elastic conductors without surfactant on SGSs (Blue) and a control substrate (Black).

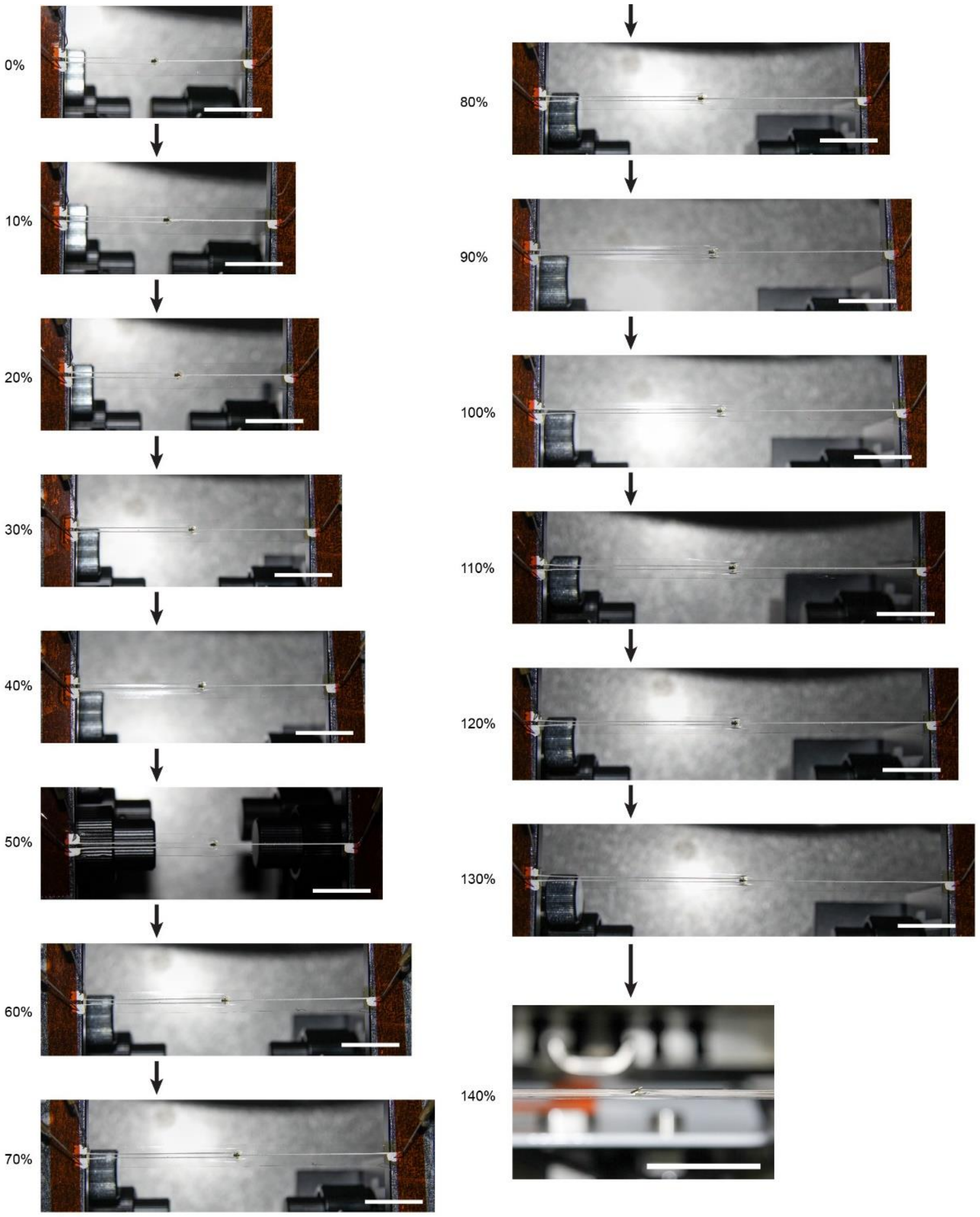

Supplementary Figure 16

Sequence of measuring stretchable single transistor. Scale bars, 2 cm.

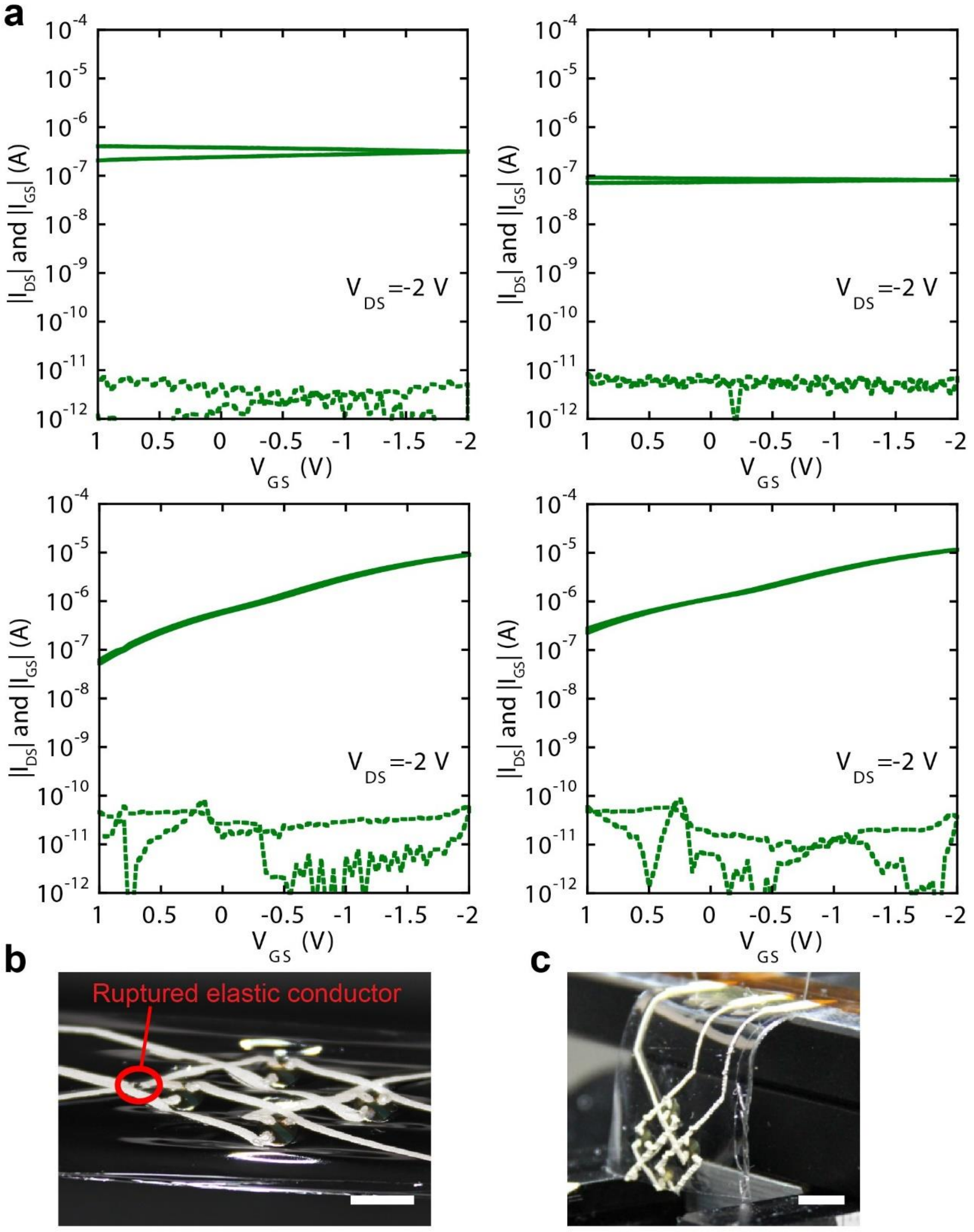

Supplementary Figure 17

Fracture modes of stretchable oraganic transistor active matrix. a, Transfer curves of the stretchable organic transistor matrix at a strain of 140%. Continuous lines: $|I_{DS}|$. Dashed lines: $|I_{GS}|$. **b,** Rupture of elastic conductor. Scale bar, 5 mm. **c,** Completely torn stretchable organic transistor active matrix. Scale bar, 5 mm.

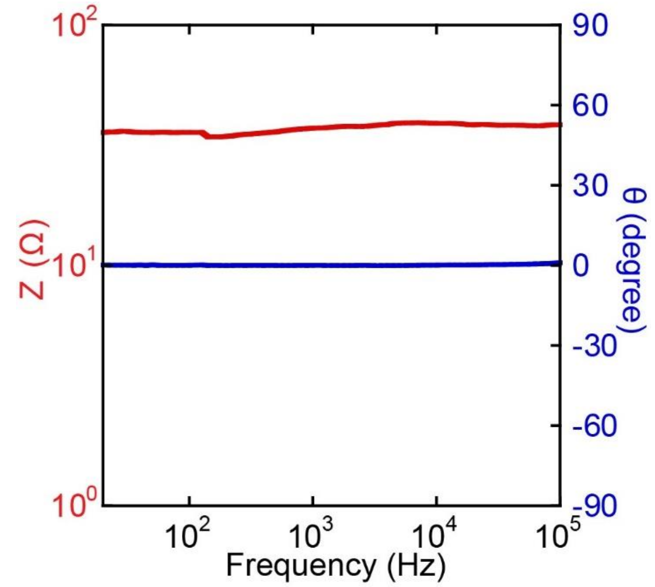

Supplementary Figure 18

Frequency dependence of elastic conductor. Red line: Z. Blue line: θ.

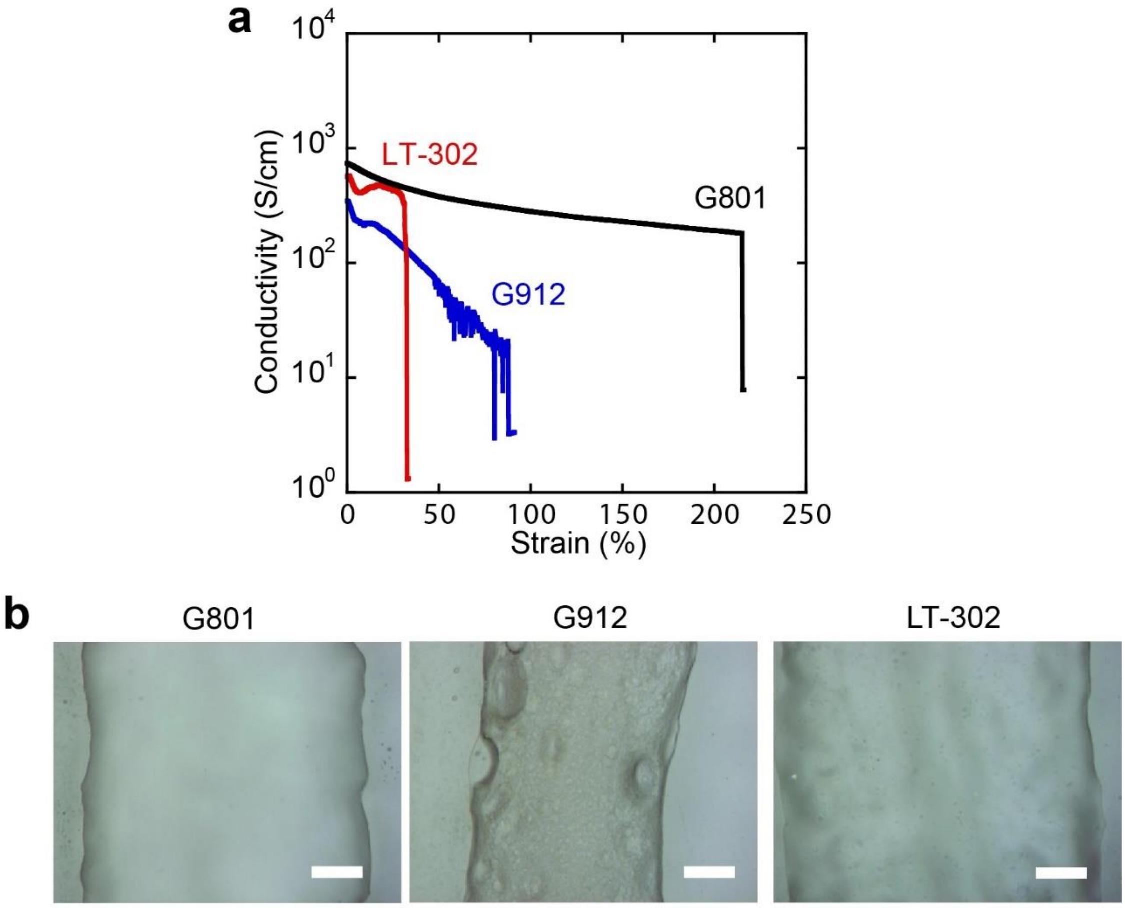

Supplementary Figure 19

Fluorine rubber effect. a, Performances of elastic conductor. **b,** Compatibility of fluorine rubbers with fluorine surfactant. Scale bars, 100 µm.
